# Association of Serum Bilirubin with Contrast-Induced Nephropathy and Future Cardiovascular Events in Patients Undergoing Coronary Intervention

**DOI:** 10.1371/journal.pone.0042594

**Published:** 2012-08-06

**Authors:** Shao-Sung Huang, Po-Hsun Huang, Tao-Cheng Wu, Jaw-Wen Chen, Shing-Jong Lin

**Affiliations:** 1 Division of Cardiology, Taipei Veterans General Hospital, Taipei, Taiwan; 2 Department of Medical Research and Education, Taipei Veterans General Hospital, Taipei, Taiwan; 3 Institute of Clinical Medicine, National Yang-Ming University, Taipei, Taiwan; 4 Cardiovascular Research Center, National Yang-Ming University, Taipei, Taiwan; 5 Institute and Department of Pharmacology, National Yang-Ming University, Taipei, Taiwan; College of Pharmacy, University of Florida, United States of America

## Abstract

**Objectives:**

Enhanced reactive oxygen species formation within the kidney following the administration of contrast media may play a key role in the development of contrast-induced nephropathy (CIN). Bilirubin has emerged as an important endogenous antioxidant molecule. This study was undertaken to determine whether bilirubin is associated with CIN and future cardiovascular events in patients undergoing coronary intervention.

**Methods:**

Totally, 544 consecutive patients received coronary intervention were enrolled. All patients were followed up for at least 3 years or until the occurrence of a major event. The primary endpoint was CIN, defined as a rise in serum creatinine (SCr) of 0.5 mg/dl or a 25% increase from the baseline value within 48 hours after the procedure. The secondary endpoint was the combined occurrence of major adverse cardiovascular events (MACE), including death, nonfatal myocardial infarction, and ischemic stroke.

**Results:**

Overall, CIN occurred in 85 (15.6%) patients. All patients were stratified into 3 groups (low/normal/high) according to the serum bilirubin levels. In a multivariate logistic analysis, the odds ratio for CIN with low-bilirubin levels relative to high-bilirubin levels was 11.82 (95% CI, 3.25–43.03). By Cox regression analysis, serum bilirubin levels was an independent predictor of MACE in patients undergoing coronary intervention (low vs. high hazard ratio 2.26; 95% CI, 1.05–4.90).

**Conclusions:**

CIN is a serious complication of coronary intervention. Higher serum bilirubin concentrations were associated with lower risk of CIN and fewer cardiovascular events. The development of interventions that promote bilirubin levels may be a potential target to reduce CIN and future MACE in patients undergoing coronary intervention.

## Introduction

Contrast-induced nephropathy (CIN) remains a serious clinical problem in the use of iodinated contrast media, which accounts for a significant number of cases of hospital-acquired acute kidney injury [1]–[3]. The risk of CIN continues to be considerable, despite the use of newer and less nephrotoxic contrast agents in high-risk patients in recent years [4]. CIN is a possible complication of coronary diagnostic and interventional procedures. Its development has been associated with increased in-hospital and long-term morbidity and mortality, prolonged hospitalization, and long-term renal impairment [5]–[7]. Proposed pathophysiologic mechanisms through which contrast administration may potentiate renal injury include oxidative stress, free radical damage, and endothelial dysfunction [8], [9]. Enhanced reactive oxygen species (ROS) generation in clinical conditions predisposing to CIN and the evidence for enhanced ROS formation following exposure to contrast media, highlight the possibility that oxidative stress may play an important role in the pathogenesis of CIN.

Bilirubin, once considered simply the metabolic end-product of heme degradation by heme-oxygenase (HO), has now emerged as an important endogenous anti-inflammatory and antioxidant molecule. It is a potent antioxidant under physiological conditions [10]. Bilirubin acts as antioxidant whether it is free or albumin bound [11], and unconjugated or conjugated [12], [13]. Accumulating evidence suggests that bilirubin may be part of a cell defense strategy in response to oxidative stress. Previous studies also indicated that bilirubin serves as a physiological antioxidant in ischemia-reperfusion [14]. Serum bilirubin may prevent experimental atherosclerosis possibly by the scavenging of oxygen radicals and by its inhibitory effects against low-density lipoprotein (LDL) oxidation [15].

Given the remarkable antioxidant, cytoprotective, and anti-inflammatory properties of bilirubin, and the critical role of oxidative stress and free radical damage in the pathogenesis of CIN, we hypothesized that higher baseline serum bilirubin level is associated with lower risk of CIN and fewer cardiovascular events in patients undergoing coronary intervention. This study was undertaken to evaluate the relationship between serum bilirubin concentrations and the incidence of CIN and the predictive role of bilirubin in clinical outcomes in patients undergoing coronary intervention.

## Methods

### Study Population

This was a retrospective study of consecutive patients with angina pectoris or acute myocardial infarction (AMI) admitted to our hospital for coronary intervention between June 2005 and December 2006. AMI was defined as detection of rise of cardiac troponin I, accompanied by either ischemic symptoms or electrocardiographic changes (including ischemic ST-segment depression, ST-segment elevation, or pathologic Q waves). Elevated cardiac troponin I was defined as a measurement exceeding the 99th percentile of the upper reference limit. Before enrollment, a detailed review of each patient’s chart was conducted to gather data on medications, smoking status, and risk factors for CIN such as age, pre-existing renal dysfunction, diabetes mellitus, volume depletion, and other comorbidities. Hypertension was defined as a systolic blood pressure ≥140 mmHg, a diastolic blood pressure ≥90 mmHg, or use of antihypertensive treatment. Diabetes mellitus was defined as fasting plasma glucose ≥126 mg/dl or use of hypoglycemic agents. Chronic kidney disease (CKD) was defined as an estimated glomerular filtration rate (eGFR) <60 ml/min/1.73 m^2^ (which roughly corresponds in the elderly to a serum creatinine >1.0 mg/dl in a woman and >1.3 mg/dl in a man). Smokers were classified as former only if they had not smoked for more than 6 months. Body mass index (BMI) was calculated by dividing the weight of the patient in kilograms by the square of the height in meters. Patients were excluded if the coronary anatomy was not suitable for percutaneous coronary intervention (PCI) or if emergent coronary artery bypass grafting (CABG) was required. Patients with a history or clinical evidence of renal failure in chronic peritoneal or hemodialytic treatment, alcoholism or chronic liver disease were also excluded. The study was approved by the research ethics committee of Taipei Veterans General Hospital.

### Laboratory Investigations

After an overnight fast ≥12 hours, blood samples were obtained from all patients. Serum levels of glucose, aspartate aminotransferase (AST), alanine aminotransferase (ALT) and lipid profiles including triglycerides, total cholesterol, high-density lipoprotein (HDL) cholesterol and LDL cholesterol were measured using a Hitachi 7600 autoanalyzer (Hitachi Ltd., Tokyo, Japan). Serum creatinine concentration (SCr) was measured at the time of admission, and every day for the following three days after contrast media exposure. All patients were stratified into three groups according to their serum bilirubin levels [16]. Patients with bilirubin levels >0.7 mg/dl (highest tertile) were defined as the high-bilirubin group (n = 182, 33.4%), those with bilirubin levels ≤0.5 mg/dl (lowest tertile) as the low-bilirubin group (n = 181, 33.3%) and those with bilirubin levels >0.5 mg/dl and ≤0.7 mg/dl as the normal-bilirubin group (n = 181, 33.3%).

### Percutaneous Coronary Intervention

Elective PCI was performed for patients with stable coronary artery disease (CAD). Primary PCI was performed for patients with ST-segment elevation AMI if they presented within 12 hours (18 hours for AMI complicated by cardiogenic shock) from the onset of symptoms. Cardiogenic shock was defined as prolonged hypotension (systolic blood pressure <90 mmHg) with evidence of decreased organ perfusion caused by severe left ventricular dysfunction, right ventricular infarction, or mechanical complications of infarction and not due to hypovolemia, bradyarrhythmias, or tachyarrhythmias. We used nonionic low-osmolality contrast media (iopromide) for all patients. No patients received sodium bicarbonate or acetylcysteine before contrast media exposure. The use of beta-adrenergic blocking agents, angiotensin-converting enzyme inhibitors, platelet glycoprotein IIb/IIIa receptor inhibitors, diuretics, or the indication to intraaortic balloon pump (IABP) or inotropic drugs support, was left to the discretion of the interventional cardiologists. After contrast media exposure, physiologic (0.9%) saline was given intravenously at a rate of 1 ml/kg/hour for 12 hours. In patients with left ventricular dysfunction (ejection fraction <40%) or overt heart failure, the hydration rate was reduced to 0.5 ml/kg/hour.

### Clinical Follow-up for End Points

All patients included in the study were followed up for at least 3 years or until the occurrence of a major adverse cardiovascular event (MACE). The primary end point was the incidence of CIN. CIN was defined as a rise of SCr of 0.5 mg/dl or a 25% increase from the baseline value within 48 hours after PCI. The secondary end point was the combined occurrence of MACE, including death, nonfatal MI, and ischemic stroke. All participants were contacted by telephone periodically and their medical records were followed up regularly. No patients dropped out of the study, and all occurrences of adverse events were recorded. Nonfatal MI was defined by a rise of cardiac troponin I with ischemic symptoms and/or characteristic electrocardiographic changes. Ischemic stroke was defined as the presence of a new neurological deficit lasting for at least 24 hours with definite evidence of a cerebrovascular accident verified by either magnetic resonance imaging or computed tomography.

### Statistical Analysis

Data were expressed as the mean ± SD for numeric variables and as the number (percent) for categorical variables. Comparisons of continuous variables between groups were performed by Student’s t-test or one-way ANOVA test. Subgroup comparisons of categorical variables were assessed by a Chi-square or Fisher’s exact test. Survival curves were generated by the Kaplan–Meier method and survival among groups was compared by use of the log-rank test. Significant variables associated with the presence of CIN in univariate analysis were entered into the multivariate regression model. Multivariate logistic regression analysis was performed to determine the independent predictors of CIN. In order to determine the independent predictors of MACE, multivariate Cox regression analysis was performed by adjusting for variables significantly associated with clinical outcomes (including traditional risk factors, AMI, cardiogenic shock, extent of CAD, left ventricular ejection fraction [LVEF], CIN, serum bilirubin levels, and medications). Goodness-of-fit was assessed by the Hosmer-Lemeshow goodness of fit test and independent variables tested for interactions and multicollinearity. Data were analyzed using SPSS software (version 17, SPSS, Chicago, Illinois, USA). A P value of <0.05 was considered to indicate statistical significance.

## Results

### Patient Characteristics

A total of 544 consecutive patients (463 males, 85%) were enrolled in this study. The mean age of our cohort was 70±11 years. Of them, 85 (15.6%) developed CIN after coronary intervention. The baseline characteristics of all patients are shown in **Table 1**. No significant differences were found between the patients with or without CIN with respect to smoking status, serum levels of lipid profiles and liver enzymes, and underlying diseases such as hypercholesterolemia and previous MI. However, patients with CIN tended to be older, were more often female, had a higher prevalence of hypertension, diabetes, and CKD, had lower LVEF, and were more likely to present with lower serum bilirubin concentrations than patients without CIN (*P*<0.001).

**Table 1:** Baseline characteristics of patients with or without CIN.

	With CIN	Without CIN	
	(n = 85)	(n = 459)	*P*
Age (years)	74.4±9.7	69.6±12.0	<0.001
Male	65 (76.5%)	398 (86.7%)	0.015
Body mass index (kg/m2)	24.2±3.4	25.1±4.1	0.059
Current smoker	27 (31.8%)	183 (39.9%)	0.159
Hypertension	68 (80.0%)	304 (66.2%)	0.012
Diabetes mellitus	55 (64.7%)	146 (31.8%)	<0.001
Hypercholesterolemia	19 (22.4%)	97 (21.1%)	0.801
Chronic kidney disease	48 (56.5%)	79 (17.2%)	<0.001
Previous MI	9 (10.6%)	77 (16.8%)	0.151
Previous stroke/TIA	14 (16.5%)	30 (6.5%)	0.002
Prior PCI/CABG	25 (29.4%)	141 (30.7%)	0.810
Lipid profiles (mg/dl)			
Triglycerides	152.5±146.1	137.6±93.2	0.370
Total cholesterol	176.1±54.1	174.0±38.0	0.745
High-density lipoprotein	45.0±13.1	44.6±11.8	0.777
Low-density lipoprotein	109.7±42.1	111.0±31.2	0.797
Fasting glucose (mg/dl)	160.8±96.4	117.4±50.5	<0.001
Creatinine (mg/dl)	2.3±1.5	1.3±0.5	<0.001
Total bilirubin (mg/dl)	0.5±0.2	0.7±0.3	<0.001
AST (U/L)	34.5±30.3	39.7±37.8	0.292
ALT (U/L)	28.6±24.1	30.3±21.3	0.524
LVEF (%)	42.6±14.7	49.1±12.6	0.004

Values are mean ± SD or number (%).

CIN: contrast-induced nephropathy; MI: myocardial infarction; TIA: transient ischemic attack; PCI: percutaneous coronary intervention; CABG: coronary artery bypass grafting; AST: aspartate aminotransferase; ALT: alanine aminotransferase; LVEF: left ventricular ejection fraction.

**Table 2** lists the angiographic and hemodynamic characteristics of patients with and without CIN. Overall, 225 patients (41%) received coronary intervention because of AMI. Patients developing CIN more frequently had AMI (*P* = 0.005), triple-vessel disease (*P* = 0.001), and cardiogenic shock (*P*<0.001). In addition, patients with CIN received a higher volume of contrast media on average during PCI than those without CIN (*P* = 0.028).

**Table 2:** Angiographic and hemodynamic characteristics of patients with or without CIN.

	With CIN	Without CIN	
	(n = 85)	(n = 459)	*P*
Stable CAD	38 (44.7%)	281 (61.2%)	
Acute myocardial infarction	47 (55.3%)	178 (38.8%)	0.005
No. of diseased vessel			
Single-vessel	13 (15.3%)	136 (29.6%)	0.006
Double-vessel	27 (31.8%)	164 (35.7%)	0.482
Triple-vessel	45 (52.9%)	159 (34.6%)	0.001
Cardiogenic shock	30 (35.3%)	17 (3.7%)	<0.001
Contrast volume (ml)	278±100	252±97	0.028

Values are mean ± SD or number (%).

CIN: contrast-induced nephropathy; CAD: coronary artery disease.

### The Effects of Bilirubin on CIN and MACE

All study subjects were successfully followed up for a mean of 41±16 months. A total of 119 MACE occurred including 92 cases of death, 13 cases of nonfatal MI, and 14 cases of ischemic stroke.

**Figure 1:**
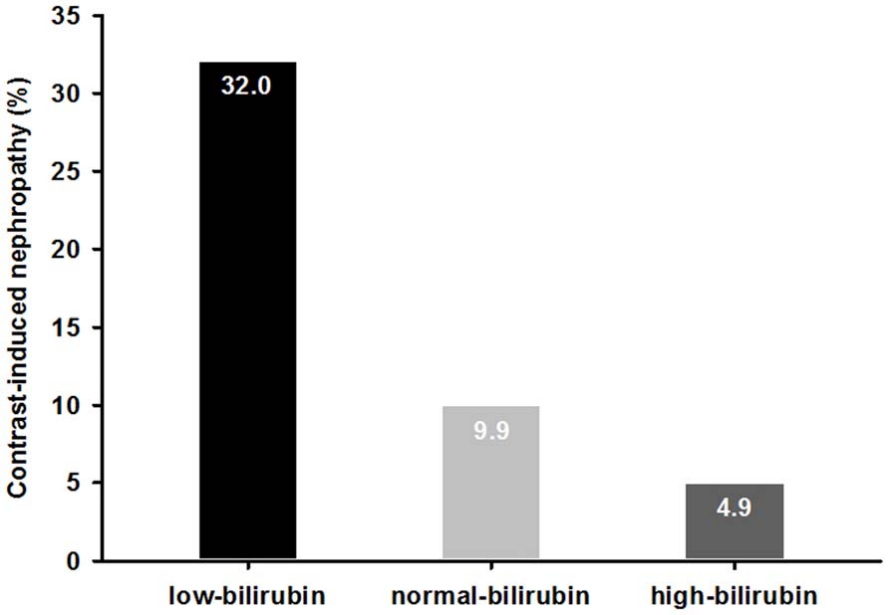
Incidence of contrast-induced nephropathy in patients stratified into low-bilirubin (≤0.5 mg/dl, n = 181), normal-bilirubin (>0.5 and ≤0.7 mg/dl, n = 181) and high-bilirubin (>0.7 mg/dl, n = 182) groups. *P*<0.001.

As shown in **Table 3**, patients in the low-bilirubin group tended to be older and had a higher prevalence of diabetes and CKD than patients in the high-bilirubin group. The incidence of CIN was significantly reduced in the high-bilirubin group (*P*<0.001), as illustrated in **Figure 1**. Patients in the high-bilirubin group had less cardiovascular death and all-cause mortality than those in the low-bilirubin group. The incidence of MACE was also significantly decreased in the high-bilirubin group compared with that in the low-bilirubin group (13.7% vs. 30.4%, *P* = 0.001), as shown in **Table 4**. In addition, higher serum bilirubin concentrations were associated with a significant decrease in the odds of MACE at discharge, 1 year and 3 years, which result in odds ratio (OR) [95% confidence interval (CI)] of 0.12 (0.02 to 0.81), 0.35 (0.13 to 0.95), and 0.20 (0.08 to 0.46), respectively.

**Table 3:** Baseline characteristics among three bilirubin groups.

	Low-bilirubin	Normal-bilirubin	High-bilirubin	
	(n = 181)	(n = 181)	(n = 182)	*P*
Age (years)	72.0±11.3	70.9±11.1	68.1±12.5	0.006
Male	150 (83%)	147 (81%)	166 (91%)	0.016
Body mass index (kg/m2)	24.4±3.5	25.0±4.5	25.4±4.0	0.077
Current smoker	106 (59%)	119 (66%)	109 (60%)	0.328
Hypertension	133 (74%)	123 (68%)	116 (64%)	0.135
Diabetes mellitus	89 (49%)	63 (35%)	49 (27%)	<0.001
Hypercholesterolemia	38 (21%)	41 (23%)	37 (20%)	0.857
Chronic kidney disease	56 (31%)	39 (22%)	32 (18%)	0.008
Previous MI	24 (13%)	32 (18%)	30 (17%)	0.491
Previous stroke/TIA	17 (9%)	18 (10%)	9 (5%)	0.159
Prior PCI/CABG	58 (32%)	55 (30%)	53 (29%)	0.832
Lipid profiles (mg/dl)				
Triglycerides	151±122	140±92	129±92	0.120
Total cholesterol	177±43	173±39	173±41	0.595
High-density lipoprotein	44±12	46±13	45±12	0.272
Low-density lipoprotein	113±36	109±30	111±33	0.584
Fasting glucose (mg/dl)	133±76	121±57	117±47	0.044
Creatinine (mg/dl)	1.6±1.1	1.4±0.8	1.3±0.5	<0.001
LVEF (%)	46.3±13.6	50.8±12.4	47.7±13.0	0.019
Contrast volume (ml)	259±98	247±77	262±115	0.309

Values are mean ± SD or number (%).

MI: myocardial infarction; TIA: transient ischemic attack; PCI: percutaneous coronary intervention; CABG: coronary artery bypass grafting; LVEF: left ventricular ejection fraction.

**Table 4:** Adverse events based on serum bilirubin levels.

	Bilirubin (mg/dl)			
	≤0.5	>0.5 and ≤0.7	>0.7	
	(n = 181)	(n = 181)	(n = 182)	*P*
Need for hemodialysis	19 (10.5%)	8 (4.4%)	5 (2.7%)	0.004
Nonfatal MI	6 (3.3%)	4 (2.2%)	3 (1.6%)	NS
Ischemic stroke	4 (2.2%)	3 (1.7%)	7 (3.8%)	NS
Cardiovascular death	28 (15.6%)	16 (8.8%)	10 (5.5%)	0.005
All-cause death	45 (24.9%)	32 (17.7%)	15 (8.2%)	<0.001
MACE (death/MI/stroke)	55 (30.4%)	39 (21.5%)	25 (13.7%)	0.001

Values are number (%).

MI: myocardial infarction; MACE: major adverse cardiovascular event.

**Figure 2:**
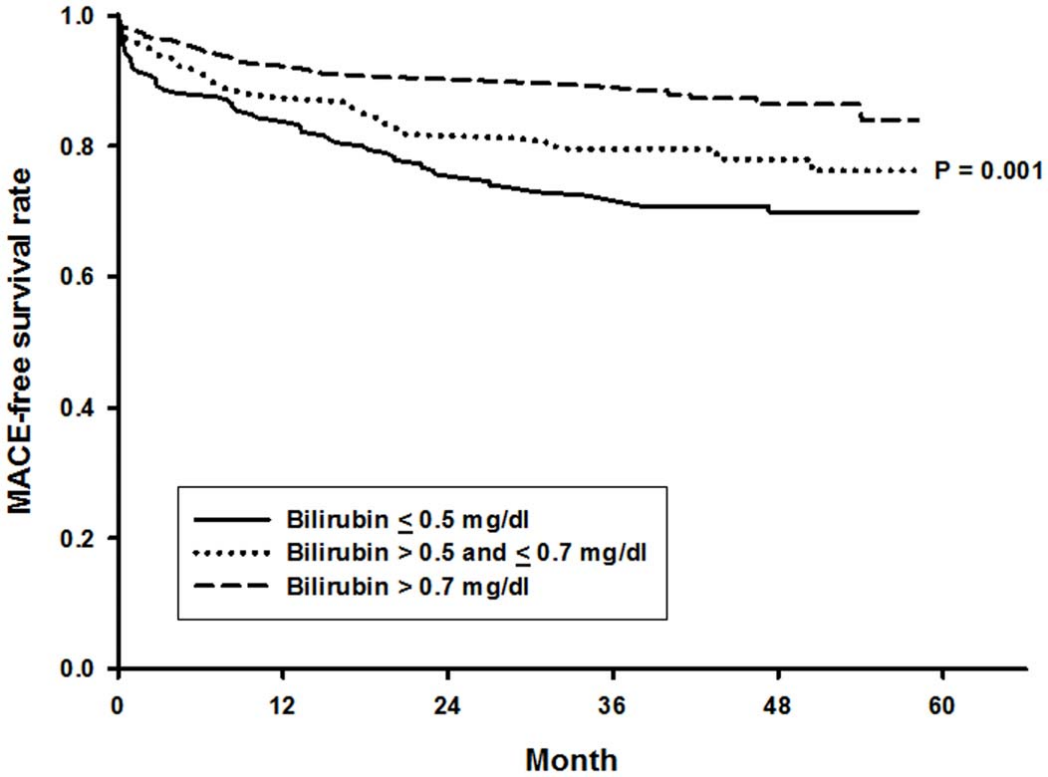
Kaplan–Meier survival curves stratified by the serum bilirubin levels. The MACE-free survival rate was significantly reduced in patients with lower serum bilirubin levels (*P = *0.001 by log-rank test).

In order to determine the relationship between survival free of adverse events and serum bilirubin levels, Kaplan–Meier survival analysis was performed. Patients with lower serum bilirubin concentrations had significantly reduced MACE-free survival rate (*P* = 0.001), as illustrated in **Figure 2**.

### Independent Correlates of CIN and Predictors of MACE

In order to investigate the association of serum bilirubin levels with CIN, multivariate logistic regression analysis was performed. Because the diagnosis of diabetes and CKD were associated with greater risk, serum levels of fasting glucose and creatinine were not entered into the multivariate model. After adjustment for age, gender, history of hypertension, diabetes, CKD and previous stroke/transient ischemic attack, AMI, cardiogenic shock, extent of CAD, LVEF, and contrast volume, the OR for CIN with normal-bilirubin levels relative to high-bilirubin levels was 4.83 (95% CI, 1.22–19.10, *P* = 0.025) and the OR for CIN with low-bilirubin levels relative to high-bilirubin levels was 11.82 (95% CI, 3.25–43.03, *P*<0.001), as shown in **Table 5**. We used the Hosmer and Lemeshow test to evaluate whether the multivariate logistic regression model is well calibrated. The test result indicated the fit is good (*P* = 0.726). In addition, there was no evidence of interactions and multicollinearity between bilirubin and other confounding factors for CIN.

**Table 5:** Multivariate logistic analysis* of risk factors for CIN.

	OR	95% CI	*P*
Contrast Volume (ml)	1.006	1.002–1.009	0.001
LVEF ≤40%	2.856	1.280–6.369	0.010
Chronic kidney disease	2.991	1.287–6.948	0.011
Diabetes mellitus	3.687	1.597–8.512	0.002
Normal vs. high bilirubin levels	4.826	1.219–19.10	0.025
Low vs. high bilirubin levels	11.82	3.245–43.03	<0.001
Cardiogenic shock	32.62	7.781–136.8	<0.001

OR: odds ratio; CI: confidence interval; LVEF: left ventricular ejection fraction.

* Adjusted for age, gender, medical history (hypertension, diabetes, chronic kidney disease, previous stroke/transient ischemic attack), acute myocardial infarction, cardiogenic shock, extent of coronary disease, left ventricular ejection fraction, contrast volume, and serum bilirubin levels.

In order to investigate the independent predictors of future MACE, multivariate Cox regression analysis was performed with factors including age, medical history (hypertension, diabetes, CKD, and smoking), AMI, cardiogenic shock, extent of CAD, LVEF, CIN, serum bilirubin concentrations, and medications (β-blockers, statins, and angiotensin-converting enzyme inhibitors). As shown in **Table 6**, serum bilirubin levels was an independent predictor of MACE in patients undergoing coronary intervention (low vs. high: hazard ratio [HR] 2.26, 95% CI 1.05–4.90, *P* = 0.038).

**Table 6:** Multivariate Cox regression analysis* of risk factors for MACE.

	HR	95% CI	*P*
Statins	0.353	0.186–0.673	0.002
β-blockers	0.507	0.290–0.889	0.018
Chronic kidney disease	1.942	1.089–3.463	0.024
Low vs. high bilirubin levels	2.263	1.046–4.897	0.038
Contrast-induced nephropathy	5.344	2.720–10.50	<0.001

HR: hazard ratio; CI: confidence interval.

* Adjusted for age, medical history (hypertension, diabetes, chronic kidney disease, smoking), acute myocardial infarction, cardiogenic shock, extent of coronary disease, left ventricular ejection fraction, contrast-induced nephropathy, serum bilirubin levels, and medications (angiotensin-converting enzyme inhibitors, β-blockers, statins).

## Discussion

The major finding of the present study indicated that patients undergoing coronary intervention with lower serum bilirubin concentrations had higher incidence of CIN and future cardiovascular events. These findings suggest that enhanced oxidative stress and insufficient anti-oxidative defense mechanism are the potential pathophysiology in patients developing CIN.

Nephropathy induced by contrast media is a significant yet underestimated problem in clinical practice. With the increasing use of contrast media in diagnostic and interventional procedures over the last 30 years, this form of nephropathy has become an important cause of hospital-acquired acute renal failure. Several risk factors for CIN have been identified. Chronic renal insufficiency, diabetes mellitus, congestive heart failure, intravascular volume depletion, and the use of a large amount of contrast agent are considered important predisposing factors [5], [7], [17]. The risk of CIN continues to be considerable, despite the use of newer and less nephrotoxic contrast agents in high-risk patients in recent years [4], and affected patients are at increased risk of morbidity and death. They may require short-term hemodialysis, which can extend their hospital stay and increase the risk of permanent impairment of renal function [18].

ROS, principally generated in outer medulla [19], may increase following the administration of contrast media, given the decline in medullary blood flow and oxygenation and the increase in tubular transport activity. Inhibition of HO-1, which suppresses ROS formation, further enhanced renal parenchymal superoxide generation following contrast media administration [20]. Patients with CKD, the most important factor predisposing to CIN, have defective antioxidant systems and increased oxidative stress [21]–[23]. ROS generation was also shown to be proportional to the amount of injected contrast media [24]. Altered antioxidant systems may further intensify contrast media-induced renal ROS formation. Enhanced ROS generation in clinical conditions predisposing to CIN and the evidence for enhanced ROS formation following exposure of contrast media, highlight the possibility that oxidative stress may play an important role in the pathogenesis of CIN. Another important factor in the pathogenesis of CIN is the induction of endothelial dysfunction and altered renal microcirculation. ROS play a pivotal role in this phenomenon by the generation of vasoconstrictors, such as isoprostane [25]. Most importantly, enhancement of ROS may reduce nitric oxide (NO) bioavailability [26]. In animal studies, administration of xanthine-oxidase inhibitor allopurinol and superoxide dismutase was found to prevent contrast-induced altered NO generation in rats [27]. ROS and NO interactions have a profound role in renal oxygenations and in the generation of CIN. Therefore, induction of antioxidants protects against the development of CIN. Goodman et al [20] suggested that the induction of HO-1 in rats subjected to a CIN model prevents the rise in superoxide and the subsequent development of tubular cell apoptosis and renal failure. In our study, we found that bilirubin, the metabolic end product of heme degradation by HO, is an independent determinant of and had an inverse correlation to CIN in patients undergoing coronary intervention.

Bilirubin has emerged as an important endogenous anti-inflammatory and antioxidant molecule. The properties of bilirubin might protect patients from cardiovascular events by limiting the development of atherosclerotic vascular disease. A number of mechanisms have been proposed for the antiatherogenic effects of bilirubin, including suppression of LDL oxidation [15], monocyte migration [28], vascular smooth muscle cell proliferation [29], and endothelial dysfunction [30]. Besides, bilirubin prevents experimental atherosclerosis possibly by scavenging oxygen radicals [15]. Although the direct pathological connection of serum bilirubin with clinical atherosclerosis has not been fully established, there is some indication that serum bilirubin could relate inversely to the development of CAD [31]. To the best of our knowledge, the present study is the first to indicate the predictive role of serum bilirubin for long-term outcomes in patients with established CAD. Our data showed that patients in the high-bilirubin group had less cardiovascular death and all-cause mortality than those in the low-bilirubin group. Higher serum bilirubin concentrations were associated with a significant decrease in the odds of MACE at discharge, 1 year and 3 years. Further large prospective studies are required to confirm our findings.

Given the remarkable antioxidant and anti-inflammatory properties of bilirubin, and the role of inflammation and oxidative stress in the pathogenesis of CIN and atherosclerosis, it would be interesting to initiate some clinical intervention that could raise serum bilirubin levels, perhaps by inducing HO [32], as a potential strategy to prevent CIN and future cardiovascular events in CAD patients undergoing coronary intervention.

### Study Limitations

There are some limitations that should be considered in this study. First, the present study included a small population at a single center. These findings should be confirmed in a large multicenter trial. Second, many confounding variables, such as age, gender, history of diabetes and CKD, AMI, cardiogenic shock, LVEF, and contrast volume were demonstrated to influence the generation of CIN. After these variables were adjusted, serum bilirubin level still independently predicted the development of CIN in patients undergoing coronary intervention. Although we are unable to determine whether hypobilirubinemia has a causative effect, the mechanisms described above may partly explain the inverse correlation between serum bilirubin concentrations and incidence of CIN following exposure of contrast media. Further studies are needed to clarify the exact interaction between bilirubin and CIN.

### Conclusions

CIN is a serious complication of coronary intervention. Higher serum bilirubin level is associated with lower risk of CIN and fewer cardiovascular events. The development of interventions that promote bilirubin levels may be a potential target to reduce CIN and future MACE in patients undergoing coronary intervention.
